# Bronchiolitis Obliterans Syndrome Following High-Dose Chemotherapy and Autologous Hematopoietic Stem Cell Transplantation in a Pediatric Patient With Neuroblastoma

**DOI:** 10.7759/cureus.102291

**Published:** 2026-01-26

**Authors:** Naomi Ogawa, Daisuke Morita, Hirokazu Morokawa, Kazutoshi Komori, Kazuo Sakashita

**Affiliations:** 1 Department of Hematology and Oncology, Nagano Children's Hospital, Azumino, JPN

**Keywords:** autologous hematopoietic stem cell transplantation, bronchiolitis obliterans syndrome, high-dose chemotherapy, pediatric case, post-transplant pulmonary complication

## Abstract

Bronchiolitis obliterans syndrome (BOS) is a chronic obstructive pulmonary disorder most often recognized as a pulmonary manifestation of chronic graft-versus-host disease after allogeneic hematopoietic stem cell transplantation (HSCT). Its occurrence following autologous HSCT (auto-HSCT) is exceedingly rare, and its clinical features and prognosis remain poorly defined. We report the case of a five-year-old girl with neuroblastoma who developed BOS after high-dose chemotherapy with thiotepa and melphalan followed by auto-HSCT. Respiratory symptoms, including dyspnea, wheeze, and oxygen desaturation, emerged almost simultaneously with hematopoietic engraftment. Computed tomography demonstrated mosaic attenuation with air trapping, and pulmonary function tests revealed peripheral airway obstruction, while infectious evaluations were entirely negative. Despite initial treatment with bronchodilators and corticosteroids, symptoms persisted beyond six weeks, leading to a diagnosis of BOS. Multimodal therapy, including methylprednisolone pulse therapy, intravenous immunoglobulin, inhaled corticosteroids, and azithromycin, resulted in the resolution of clinical symptoms and discontinuation of oxygen supplementation, although radiographic abnormalities remained. This case suggests that, although BOS is generally considered irreversible after allo-HSCT, BOS following auto-HSCT in children may follow a more reversible course, underscoring the importance of timely diagnosis and therapeutic intervention, and supporting the concept that the underlying mechanisms of BOS may differ between allo- and auto-HSCT.

## Introduction

Hematopoietic stem cell transplantation (HSCT) can be broadly categorized into allogeneic transplantation, in which stem cells are derived from a donor, and autologous transplantation, in which a patient’s own stem cells are reinfused after high-dose chemotherapy. While allogeneic HSCT is associated with alloimmune complications such as graft-versus-host disease (GVHD), auto-HSCT does not involve alloimmune responses and is generally considered to carry a lower risk of immune-mediated organ injury.

Bronchiolitis obliterans syndrome (BOS) is a chronic obstructive pulmonary disorder most often recognized as a pulmonary manifestation of chronic GVHD after allogeneic HSCT [1]. It is characterized by progressive small-airway obstruction caused by fibrotic remodeling of the terminal and respiratory bronchioles and is associated with significant morbidity and mortality. Cohort studies have reported an overall prevalence of approximately 5%-10% among HSCT recipients, with a five-year survival rate as low as 10%-20% once BOS develops, highlighting its substantial mortality and poor long-term prognosis [2,3].

In the setting of allogeneic HSCT, BOS is primarily driven by alloimmune mechanisms related to chronic GVHD, in which donor-derived T cells initiate immune-mediated injury to the airway epithelium through T cell receptor-dependent recognition of alloantigens, resulting in progressive fibrotic remodeling of the small airways [1]. Although these alloimmune processes are central to GVHD-related BOS, recent studies have shown that BOS may also develop from non-alloimmune immune dysregulation and epithelial injury, integrating cytotoxic exposures, noninfectious inflammatory injury, and aberrant repair of the distal airways [4-6].

Given these findings, BOS can occur even after auto-HSCT through non-alloimmune mechanisms. In this context, high-dose chemotherapy may induce cytotoxic injury to the bronchial epithelium, resulting in the release of damage-associated molecular patterns (DAMPs) from injured airway epithelial cells. During subsequent hematopoietic engraftment, an imbalanced process of immune reconstitution within the autologous immune system may amplify these danger signals, thereby triggering inflammatory responses in the distal airways. Although only a few such cases have been reported in adults, this condition should nevertheless be recognized as a possible post-transplant complication. Previous reports have suggested that its pathogenesis may differ from that of BOS following allogeneic HSCT, potentially resulting in distinct clinical courses and outcomes [7,8]. Importantly, these non-alloimmune mechanisms and their clinical implications remain poorly defined in pediatric patients, as BOS following auto-HSCT has not been previously reported in the pediatric literature. Consequently, important gaps remain regarding the timing of onset, diagnostic approach, and potential reversibility in this population.

The 2014 National Institutes of Health (NIH) consensus criteria standardized the diagnostic framework for BOS following HSCT, emphasizing physiological airflow obstruction, characteristic radiologic findings, and the exclusion of alternative causes [9]. Because lung biopsy carries significant procedural risk and has limited diagnostic yield, the NIH criteria also note that BOS can be diagnosed based on noninvasive assessments in appropriate clinical contexts, reflecting the practical challenges of obtaining histologic confirmation, especially in pediatric and transplant populations. Furthermore, in pediatric patients, adult diagnostic criteria cannot be directly applied without modification. Recent pediatric-focused reviews have pointed out that conventional forced expiratory volume in one second (FEV₁)-based thresholds may fail to detect early disease, and that indices sensitive to small-airway dysfunction and characteristic computed tomography (CT) findings are crucial for early recognition and intervention [10].

Here, we report a pediatric case of BOS occurring after high-dose chemotherapy and subsequent auto-HSCT for neuroblastoma, in which early recognition and prompt therapeutic intervention led to clinical and functional recovery, an outcome distinctly different from the typically irreversible course of BOS following allogeneic HSCT. We further discuss diagnostic considerations and potential pathobiological mechanisms underlying this rare presentation.

## Case presentation

A five-year-old girl was diagnosed with left adrenal neuroblastoma (poorly differentiated, MYCN amplification positive, stage M) at age four, following presentation with abdominal distension and anemia. She received three courses of induction chemotherapy consisting of cyclophosphamide, vincristine, pirarubicin, and cisplatin, followed by two courses of ICE therapy consisting of ifosfamide, carboplatin, and etoposide. Thereafter, five courses of consolidation chemotherapy were administered, followed by high-dose chemotherapy with thiotepa and melphalan, and subsequent auto-HSCT. Hematopoietic engraftment was confirmed on day 10 after transplantation.

Shortly after engraftment was confirmed, the patient developed dyspnea with nocturnal oxygen desaturation, and within several days, productive cough and expiratory wheezing became evident. Chest radiography excluded pneumonia and congestive heart failure. A comprehensive infectious workup, including respiratory film array and tests for cytomegalovirus, fungi, *Bordetella pertussis*, tuberculosis, *Chlamydia pneumoniae*, and *Mycoplasma pneumoniae*, was entirely negative. Acute bronchiolitis was initially suspected, and she was managed with supplemental oxygen.

**Figure 1:**
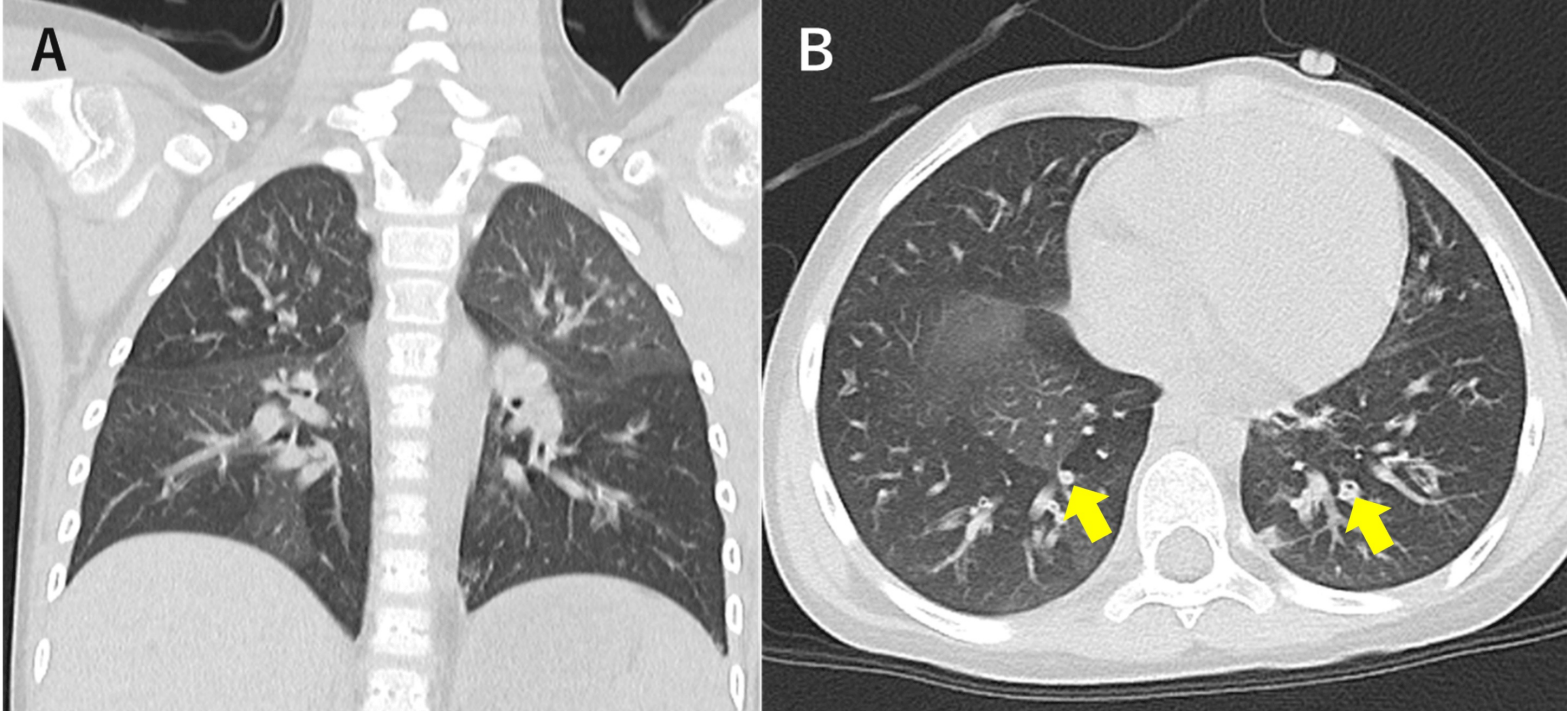
Chest CT demonstrating mosaic attenuation with air trapping Coronal (A) and axial (B) chest CT obtained on day 25 after autologous HSCT show a mosaic attenuation pattern with air trapping, corresponding to areas of bronchial wall thickening (arrows). No ground-glass opacities or centrilobular nodules are observed CT: computed tomography; HSCT: hematopoietic stem cell transplantation

Despite supportive care, respiratory symptoms persisted for over two weeks, with progressive expiratory wheezing and worsening oxygen desaturation. Chest CT on day 25 demonstrated mosaic attenuation with evident air trapping corresponding to areas of bronchial wall thickening, without centrilobular nodules or ground-glass opacities (Figure 1).

**Figure 2:**
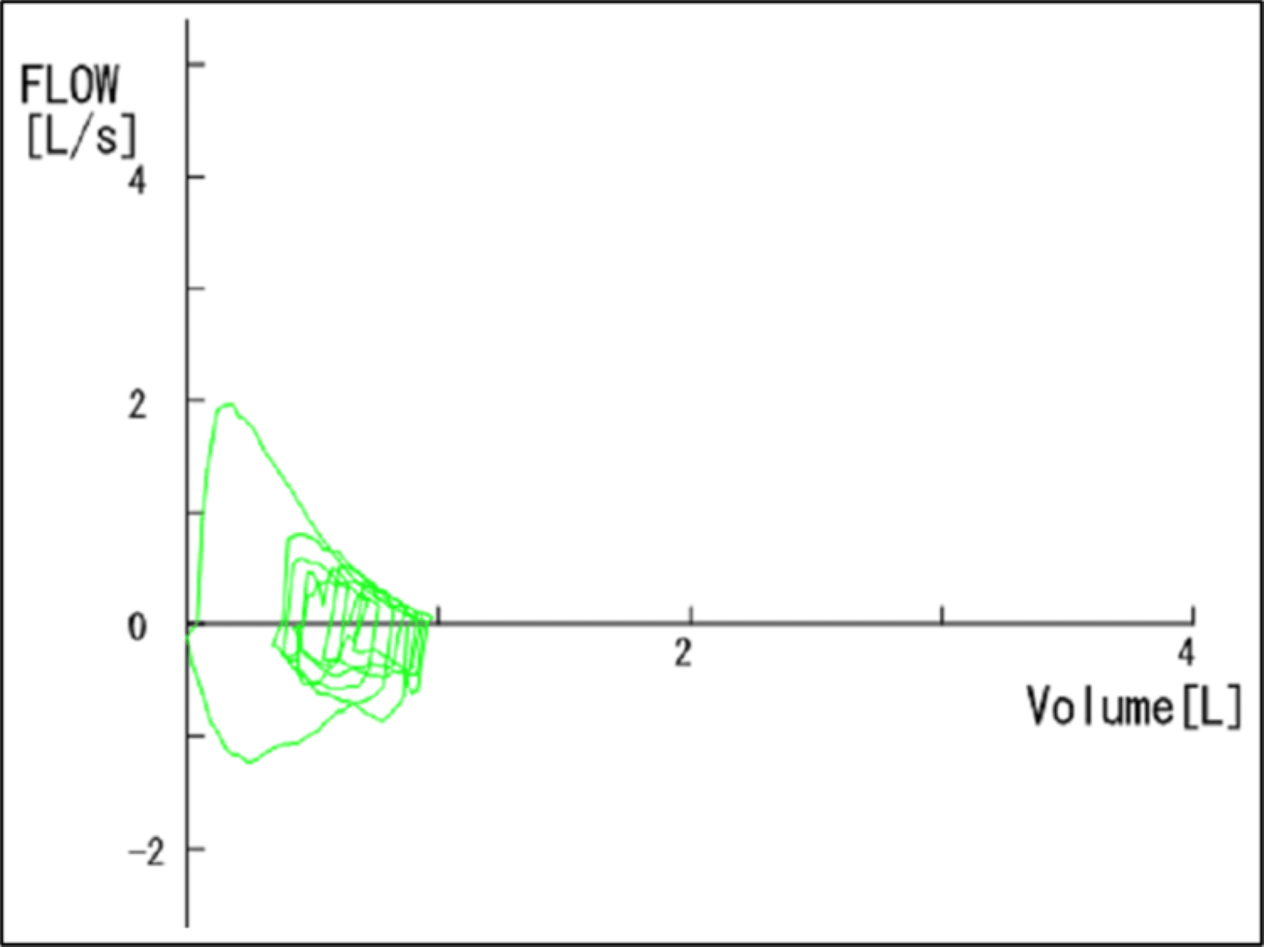
Flow-volume curve demonstrating peripheral airway obstruction The flow-volume curve obtained two weeks after symptom onset demonstrates a concave expiratory pattern, indicating impaired small-airway function

Serum KL-6 and SP-D were within normal limits. Serum ferritin was elevated at 1,875 ng/mL, and soluble interleukin-2 receptor (sIL-2R) was also increased at 624 U/mL. Pulmonary function tests showed an FEV₁ of 88.2% predicted, with other parameters within normal limits. However, the flow-volume curve demonstrated a concave pattern, and maximal expiratory flow at 25% of forced vital capacity (V25) was markedly reduced (0.33 L/s), both indicative of peripheral airway obstruction (Figure 2, Table 1).

**Table 1:** Early obstructive airway change revealed by decreased V25 with preserved FEV1 Pulmonary function test performed two weeks after symptom onset shows a markedly decreased V25 (0.33 L/s) despite normal FEV1, indicating early small-airway obstruction characteristic of bronchiolitis obliterans syndrome. Percent predicted values were calculated using the CHESTAC pulmonary function testing system (CHESTAC version 6.22.04; Chest M.I., Inc., Tokyo, Japan), with the predefined prediction set Pred02 based on established Japanese normative data FEV1: forced expiratory volume in one second

Flow-volume curve	Measured	Predicted	% Predicted
Peak flow (L/s)	1.98	1.53	129%
V50 (L/s)	1.02	1.27	80%
V25 (L/s)	0.33	0.9	36%

Although the findings were suggestive of bronchiolitis obliterans, diagnostic criteria require chronicity of symptoms and exclusion of asthma. Accordingly, inhaled β₂-agonists, oral montelukast, and systemic corticosteroids (prednisolone 2 mg/kg/dose intravenously every eight hours) were initiated.

**Figure 3:**
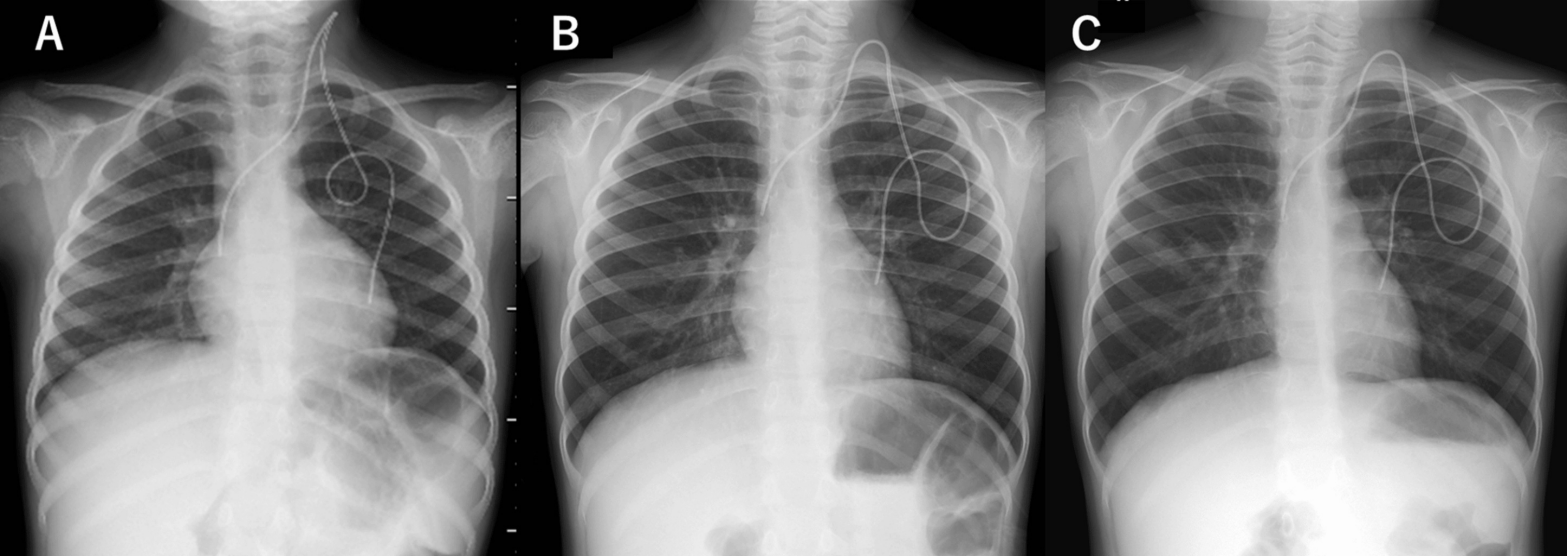
Progressive hyperinflation persisting over six weeks despite treatment with bronchodilators and corticosteroids (A) Chest radiograph prior to onset shows no remarkable hyperinflation. (B) Two weeks after symptom onset, hyperlucency, diaphragmatic flattening, and a barrel-shaped thorax are observed. (C) Six weeks after onset, hyperinflation findings had progressed despite treatment with bronchodilators and corticosteroids

**Figure 4:**
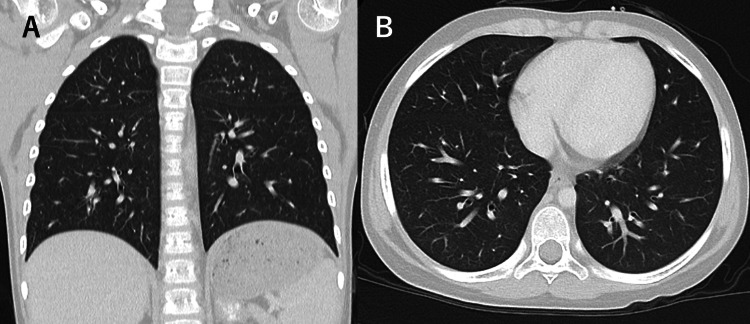
Chest CT showing progression of air trapping six weeks after onset Coronal (A) and axial (B) chest CT obtained six weeks after onset demonstrates progression of air trapping, with diffuse hyperinflation involving almost the whole lung compared with the earlier study CT: computed tomography

The therapeutic response was minimal, and obstructive symptoms progressed over six weeks. At that point, the chest radiograph showed more evident hyperlucency with a flattened diaphragm, barrel-shaped thorax, and a narrow cardiac shadow (Figure 3). Chest CT demonstrated expansion of air trapping lesions (Figure 4). In addition, previously elevated serum ferritin and sIL-2R levels remained high at that time (1,709 ng/mL and 551 U/mL, respectively).

**Figure 5:**
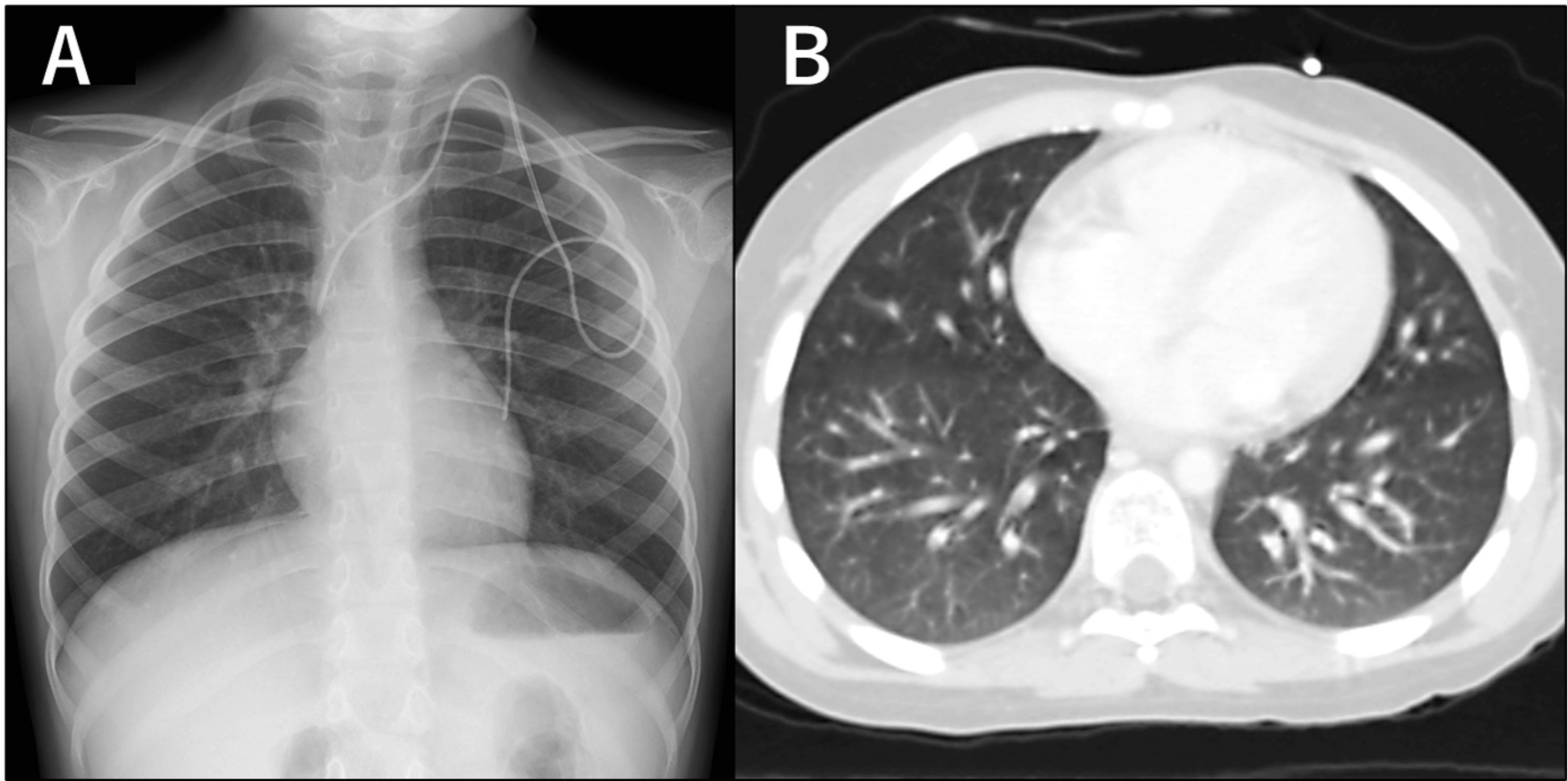
Radiologic improvement with minimal residual abnormalities after multimodal therapy (A) Chest radiograph obtained three months after initiation of therapy shows marked improvement of hyperinflation, with only minimal residual overinflation remaining. (B) Chest CT demonstrates near-complete resolution of previous air trapping, with predominantly normal lung fields CT: computed tomography

**Figure 6:**
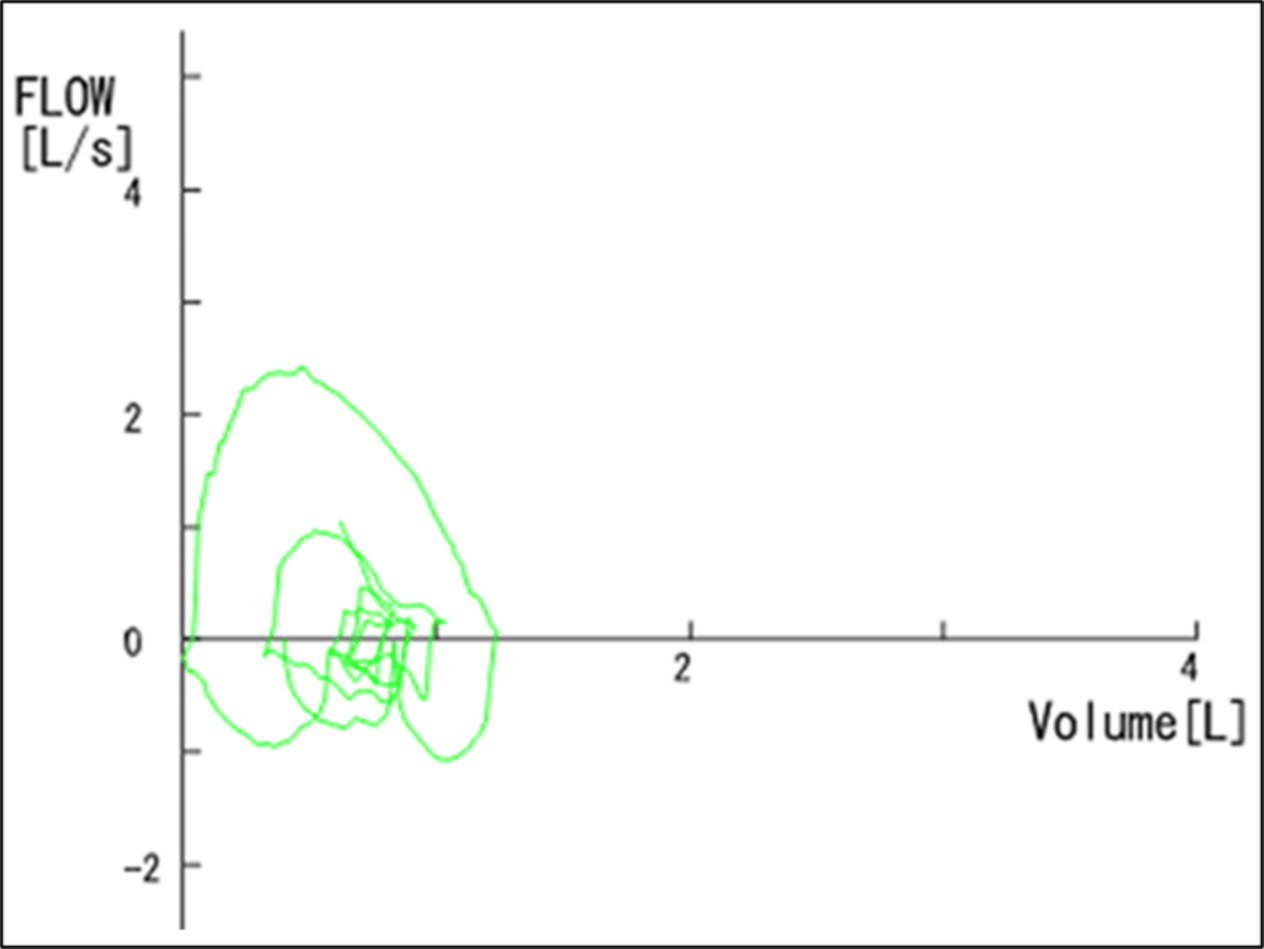
Improved flow-volume curve after multimodal therapy The flow-volume curve demonstrates a convex expiratory pattern, contrasting with the previously concave curve, indicating normalization of small-airway function following multimodal therapy

Taken together, these findings established the diagnosis of BOS. Methylprednisolone pulse therapy (30 mg/kg/day for three days monthly) was initiated in combination with intravenous immunoglobulin, inhaled corticosteroids, and azithromycin. At three months after diagnosis, although mild radiographic abnormalities persisted (Figure 5), clinical symptoms had resolved, pulmonary function had recovered (Figure 6, Table 2), and supplemental oxygen was no longer required. At nine months after diagnosis, the patient remained asymptomatic without treatment, and all objective pulmonary assessments had completely normalized (Table 3).

**Table 2:** Pulmonary function test after therapy showing normalization of V25 indicating recovery of small airway function Pulmonary function testing performed three months after diagnosis demonstrates marked improvement of flow parameters. V25 has returned to normal values, confirming recovery of peripheral airway function. Percent predicted values were calculated using the CHESTAC pulmonary function testing system, with the predefined prediction set Pred02 based on established Japanese normative data

Flow-volume curve	Measured	Predicted	% Predicted
Peak flow (L/s)	2.42	1.56	155%
V50 (L/s)	2.20	1.30	169%
V25 (L/s)	1.46	0.91	160%

**Table 3:** Summary of the clinical course and key findings This table summarizes the chronological clinical course, including key clinical findings, diagnostic investigations, therapeutic interventions, and outcomes during follow-up HSCT: hematopoietic stem cell transplantation; BOS: bronchiolitis obliterans syndrome; CT: computed tomography; PFT: pulmonary function test; sIL-2R: soluble interleukin-2 receptor; mPSL: methylprednisolone; IVIG: intravenous immunoglobulin

Timeline		Clinical findings	Investigations	Interventions	Outcome
-23 days	High-dose chemotherapy	-	-	-	-
-12 days	Autologous HSCT	-	-	-	-
-1 day	Neutrophil engraftment	-	-	-	-
0	Onset	Dyspnea, cough, O_2_ desaturation	Chest X-ray: no hyperinflation	Supportive care, Supplemental oxygen	No improvement
+3 to 5 days	Bronchiolitis under consideration (initial working diagnosis)	Expiratory wheezing	Comprehensive infectious work-up: negative	Supportive care, Supplemental oxygen	Progressive deterioration
+2 weeks	During exclusion of bronchial asthma	Progressive expiratory wheezing	Chest X-ray: hyperinflation; chest CT: mosaic attenuation; PFT: obstructive changes; ferritin and sIL-2R: elevated	Inhaled β2-agonist, oral montelukast, systemic corticosteroids, supplemental oxygen	No improvement
+6 weeks	Diagnosis of BOS	Persistent wheezing and oxygen dependence	Chest X-ray: progressive hyperinflation; chest CT: diffuse air trapping; PFT: decline; ferritin and sIL-2R: persistent elevation	mPSL pulse therapy (monthly), IVIG, inhaled corticosteroids, oral azithromycin	Improvement
+3 months after diagnosis	-	No symptoms	Chest X-ray: improving hyperinflation; chest CT: near complete resolution; PFT: normalized	No treatment	Resolution of symptoms
+9 months after diagnosis	-	No symptoms	Chest X-ray: normalized; PFT: normalized	No treatment	-

As transplantation and high-dose chemotherapy were considered likely factors contributing to the onset of BOS, the planned second course of high-dose chemotherapy was withheld. Subsequently, the patient underwent adrenal tumor resection, followed by proton beam irradiation and anti-GD2 antibody therapy.

## Discussion

Although BOS is generally recognized as a pulmonary manifestation of chronic GVHD after allogeneic HSCT, it is also well established that BOS can be driven by various forms of immune dysregulation, not limited to alloimmunity [4-6]. Immune reconstitution after auto-HSCT, marked by delayed recovery of CD4⁺ T and B cells, early natural killer cell predominance, and release of DAMPs from epithelial injury, may trigger transient immune overactivation in the distal airways [11,12]. Therefore, such immune dysregulation observed after auto-HSCT provides a rationale for considering BOS as a possible complication in this setting.

To date, only three cases of BOS after auto-HSCT have been reported: two in lymphoma and one in multiple myeloma [7,8]. BOS has also been described following chemotherapy alone [13-15]. Moreover, BOS has been reported in association with molecular targeted agents such as imatinib [16]. In these reports, in addition to direct epithelial injury of the terminal bronchioles, excessive infiltration of immune cells, such as lymphocytes, histopathologically demonstrated in some cases, was considered pathogenic.

In our case, respiratory symptoms developed almost simultaneously with hematopoietic engraftment and gradually progressed to obstructive impairment. Notably, ferritin and sIL-2R levels were elevated and remained persistently high for more than six weeks after onset. Elevated ferritin is generally regarded as a marker of macrophage-driven immune activation, whereas increased sIL-2R reflects activation of T cells and natural killer cells. The concurrent and sustained elevation of these biomarkers suggests persistent immune activation involving both innate and adaptive immune pathways, indicative of dysregulated immune reconstitution after auto-HSCT. Taken together, the clinical course and immunologic findings in this case are consistent with an immune-mediated mechanism contributing to airway injury in BOS, rather than isolated direct toxicity, and support the concept of post-transplant immune dysregulation as a central component of its pathogenesis.

Respiratory complications after auto-HSCT require broad differential diagnoses, including infection, drug-induced interstitial pneumonia, capillary leak syndrome, and heart failure [17]. When obstructive features are present, acute bronchiolitis and bronchial asthma should also be considered. As diagnostic criteria for BOS require poor responsiveness to bronchodilators for asthma and the persistence of symptoms for at least six weeks, a period of observation is often necessary [1,9]. Although histological confirmation of BOS is theoretically possible, lung biopsy is of limited practical utility due to the scattered distribution of pathological changes and its invasive nature [1]. Therefore, CT imaging and pulmonary function tests play a central role in diagnosis. Importantly, in pediatric BOS, FEV₁ -a key diagnostic criterion in adults- may remain within normal limits early in the disease course, making it essential to detect early markers of peripheral airway obstruction such as V25 in pulmonary function tests [10]. To facilitate timely recognition of BOS, it is essential to consider the disease early, perform chest CT and pulmonary function tests, and assess the response to bronchodilators with or without corticosteroids to differentiate BOS from asthma.

Regarding prognosis, BOS after allogeneic HSCT is typically irreversible due to chronic airway inflammation and fibrosis driven by GVHD, leading to significant mortality [2,3]. In contrast, the prognosis of BOS following high-dose chemotherapy and subsequent auto-HSCT remains poorly defined owing to its rarity. However, the underlying pathogenesis is thought to differ from that of allogeneic HSCT, with transient drug-related epithelial injury and immune fluctuation implicated, suggesting that the disease course may be more reversible. Indeed, a reported case of multiple myeloma after auto-HSCT demonstrated clinical improvement with systemic corticosteroids and bronchodilators [8], while other reported adult cases have followed an irreversible and fatal course (Table 4) [9].

**Table 4:** Reported cases of BOS after autologous HSCT, including the present case This table summarizes previously published cases of BOS following autologous HSCT, including patient demographics, primary disease, transplant characteristics, conditioning regimens, time to BOS diagnosis, diagnostic investigations, treatments, and clinical outcomes. The present case is included for comparison BOS: bronchiolitis obliterans syndrome; HSCT: hematopoietic stem cell transplantation; BMT: bone marrow transplantation; PBSCT: peripheral blood stem cell transplantation; BU: busulfan; CPA: cyclophosphamide; TBI: total body irradiation; MEL: melphalan; TEPA: thiotepa; PFT: pulmonary function test; CT: computed tomography; mPSL: methylprednisolone; IVIG: intravenous immunoglobulin

Study	Age/sex	Primary disease	Transplant type	Conditioning regimen	Time to BOS diagnosis	Diagnostic investigation	Treatment	Outcome
Paz et al. [7]	34/F	Hodgkin’s lymphoma	Autologous BMT	BU (16 mg/kg), CPA (120 mg/kg)	90 days	PFT: yes; CT: no; Biopsy: yes	β2-agonists, systemic corticosteroids, theophylline	Died
	41/F	Hodgkin’s lymphoma	Autologous BMT	TBI (1,200 rad) CPA (60 mg/kg)	243 days	PFT: yes; CT: yes; Biopsy: yes	Systemic corticosteroids, theophylline, azathioprine	Died
Alonso et al. [8]	67/M	Multiple myeloma	Autologous PBSCT	MEL (200 mg/m^2^)	84 days	PFT: yes; CT: yes; Biopsy: no	β2-agonists, systemic corticosteroids	Complete recovery
Present case	5/F	Neuroblastoma	Autologous PBSCT	MEL (50 mg/m^2^) TEPA (200 mg/m^2^)	54 days	PFT: yes; CT: yes; Biopsy: no	mPSL pulse therapy, IVIG, inhaled corticosteroids, oral azithromycin	Complete recovery

Prognosis may also differ between children and adults. Pediatric BOS after auto-HSCT has not been previously reported, and its prognosis remains unknown; nevertheless, in postinfectious BOS, children have been shown to have a greater potential for symptomatic improvement than adults [18]. In our case, multimodal therapy, including methylprednisolone pulse therapy, was initiated promptly after diagnosis. The observed clinical course, differing from the typically irreversible nature of BOS following allogeneic HSCT, suggests that early therapeutic intervention may be beneficial in pediatric BOS following auto-HSCT. Further accumulation of similar cases and long-term follow-up is needed to clarify outcomes in this population.

## Conclusions

This case highlights that BOS, while most commonly encountered as a pulmonary manifestation after allogeneic HSCT, should also be considered a potential complication even after auto-HSCT to facilitate timely diagnosis. Although BOS following allogeneic HSCT is generally regarded as irreversible, BOS following auto-HSCT in children may exhibit a more reversible course, likely reflecting distinct pathogenic mechanisms, underscoring the importance of early recognition and therapeutic intervention. Future studies could explore early detection strategies through serial pulmonary function testing and imaging, together with immunologic profiling during immune reconstitution, to better characterize airway injury following auto-HSCT in pediatric patients.
